# Apps for Promoting Children’s Oral Health: Systematic Search in App Stores and Quality Evaluation

**DOI:** 10.2196/28238

**Published:** 2022-06-06

**Authors:** Teresa C Y Ho, Colman McGrath, Cynthia K Y Yiu, Gillian H M Lee

**Affiliations:** 1 Paediatric Dentistry & Orthodontics Faculty of Dentistry University of Hong Kong Hong Kong China (Hong Kong); 2 Applied Oral Sciences and Community Dental Care Faculty of Dentistry University of Hong Kong Hong Kong China (Hong Kong)

**Keywords:** apps, oral health, evidence-based, oral hygiene, children

## Abstract

**Background:**

Increasingly, mobile apps are being used to promote oral care. Many of them are aimed at children.

**Objective:**

This study aimed to systematically search and evaluate apps that promote oral care and hygiene for children.

**Methods:**

A broad search strategy (13 keywords) was developed to identify apps from Apple’s App Store and the Google Play Store in April 2019. After reviewing the apps’ titles and summaries, potentially relevant apps were downloaded for viewing. The quality of the apps that met the inclusion criteria was assessed by the Health on the Net Foundation Code of Conduct (HONcode) criteria for medical and health websites and the Scientific Basis of Oral Self-care (SBOSC).

**Results:**

More than 3000 Apps were identified and 54 relevant apps informed the review. The quality of the apps according to the HONcode criteria was generally low. The mean HONcode score was 1.8/8.0. One-quarter of the apps had a HONcode score of 0 (14/54, 26%). The SBOSC score of the apps was evaluated based on a 6-point scale. The mean SBOSC score was 1.5/6.0; 19% (10/54) of the apps had a score of 0. There was a significant and positive correlation between HONcode and SBOSC scores (*r*=0.37; *P*<.01). More recently uploaded apps had significantly higher HONcode scores (*P*<.05).

**Conclusions:**

There are many apps aiming to promote oral self-care among children. The quality and scientific basis of these apps are low. Newer apps are of higher quality in terms of scientific basis. There is a need to ensure high-quality and evidence-based apps are available. The effectiveness of apps in terms of oral care and clinical outcomes among children needs to be evaluated.

## Introduction

Among all health problems that may be experienced during childhood, oral disease remains the most common [1]. A systematic analysis of the global burden of oral diseases has identified that untreated dental caries (tooth decay) among young children can cause considerable pain and suffering and impacts their quality of life, families, and communities [2]. Oral diseases are multifactorial infectious diseases and dental plaque (bacteria) plays a key role in their pathogenesis [3]. Thus, in preventing oral disease, a key focus has been on controlling dental plaque by improving oral hygiene (ie, toothbrushing) [4]. Cochrane systematic reviews are leading sources of scientific evidence to help people (both patients and clinicians) make better-informed decisions about their oral care. Several Cochrane systematic reviews have identified that toothbrushing with fluoridated toothpaste is the mainstay to prevent dental caries among children [5,6] and has minimal side effects [7].

Not surprisingly, the practice of toothbrushing is a cornerstone of oral health promotion activities, particularly among children [8]. Traditionally, toothbrushing has been promoted through conventional education programs (eg, lectures, leaflets, and posters). However, evidence of their effectiveness is questionable, particularly for long-term behavioral change and clinical outcomes [9]. Apps are increasingly used in health care and health informatics in recent years [10-12]. Promising results have been generated from narrative and systematic searches and reviews of apps for promoting mental health, physical health, and lifestyle behaviors [13-16]. In more recent times, there has been growing recognition of the potential use of apps for oral health, especially in promoting oral hygiene, and among children [17,18]. Web-based health information may not be reliable, and the quality of the knowledge delivered is not guaranteed to be high. Fallacious information could have a harmful effect on children using the apps. To date, a systematic search and review of apps for oral care is lacking, and the quality and scientific basis of such apps has not been considered. This study aimed to systematically search for and review apps for oral care aimed at young children to determine their profile characteristics, quality, and scientific basis. In addition, this study aimed to determine the relationship between the quality and scientific basis of the apps and the association between app characteristics with quality and scientific basis.

## Methods

### Data Search Strategy and Identification of Apps

**Figure 1:**
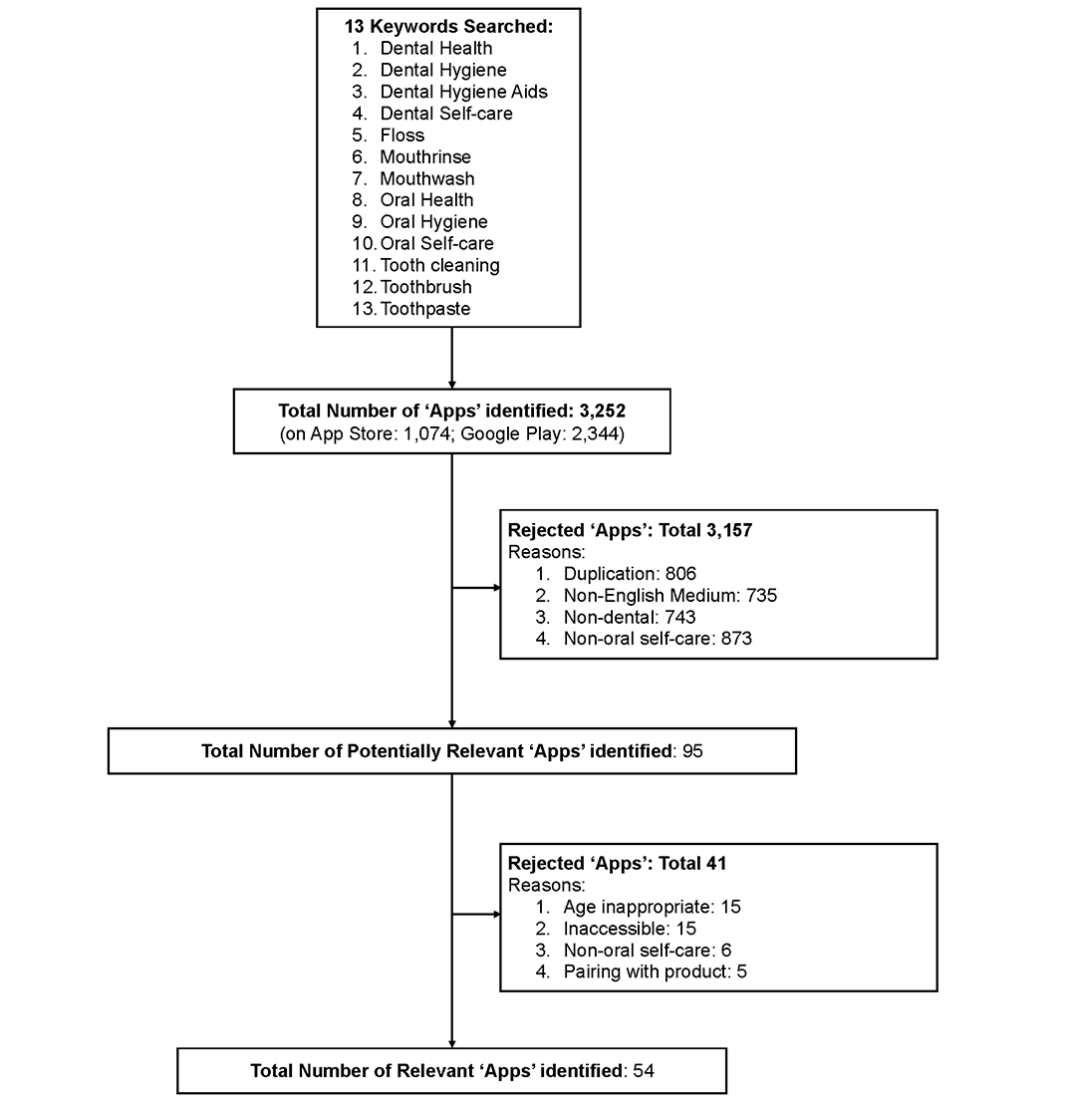
Flowchart for the Apps selection process.

A search of the Apple App Store and Google Play Store was conducted in April 2019 to identify apps designed for promoting oral self-care among children. A total of 13 oral self-care–related keywords were chosen for the search (Table 1). Screenshots of the titles and descriptions of the apps were obtained and reviewed to identify potentially relevant apps (first screening). Criteria for rejection included (1) duplicated apps, (2) non–English-language apps, (3) non–dental-related and non–oral health–related apps, and (4) non–oral self-care–related apps (Figure 1). Potentially relevant apps were downloaded and reviewed to identify relevant apps to inform the review. Criteria for rejection include (1) age-inappropriate apps (ie, age not rated as 3+ or 4+ years), (2) inaccessible apps, (3) apps requiring pairing with products to use, and (4) non–oral self-care–related apps (Figure 1). A total of 2 independent assessors conducted the search and assessments, and agreement was determined using the Cohen kappa statistic (κ=0.836). Where disagreement occurred, it was resolved with a third rater.

**Table 1:** Descriptive information on the apps reviewed (N=54).

Category of information		Apps, n (%)
**Age rating in years**		
	3+	18 (33)
	4+	36 (67)
**Compatibility**		
	Android only	18 (33)
	Apple	36 (67)
**Price**		
	Free	42 (78)
	Not free	12 (22)
**Star rating**		
	≥4	40 (74)
	<3	14 (26)
**Last upload in years**		
	≤2	41 (76)
	>2	13 (24)
**Developer**		
	Company	26 (48)
	Individual	28 (52)

### Assessment of Apps (Data Collection)

Profile information (descriptive) on the apps was obtained, including (1) age rating, (2) compatibility, (3) price, (4) star rating (rating on app platforms), (4) period of last update, and (5) developer.

All identified relevant apps were assessed for (1) Health on the Net Foundation Code of Conduct (HONcode) and (2) Scientific Basis of Oral Self-care (SBOSC) scores. The HONcode assesses the reliability and credibility (validity) of medical and health information on the internet and social media [19]. The 8 criteria of the HONcode are (1) authority (author credentials and qualifications), (2) complementarity (information supporting, but not replacing, patient–health care professional relationships), (3) privacy (anonymous and confidential use of users’ personal data), (4) attribution (references to the sources of published information and when they were last updated), (5) justifiability (balanced claims supported with references to scientific information), (6) transparency (contact information of authors provided), (7) financial disclosure (identifiable funding source), and (8) advertising policy (details about advertising on the site and distinction from editorial content). A score for each app was derived based on the 8 criteria of HONcode (with information absent scoring 0 and information present scoring 1 for each attribute). HONcode scores could range from 0-8.

The SBOSC was derived from the guidelines of the Childsmile program for oral self-care [20]. The program was based on the scientific basis of dental health education. The following 6 factors were considered: (1) choice of toothbrush (size and bristle), (2) use of fluoride toothpaste (fluoride concentration and amount), (3) brushing time (frequency, timing, and duration), (4) adult supervision, (5) brushing techniques, and (6) other advice for the prevention of caries. A score for each app was derived based on the 6 criteria of SBOSC; a score of 0 is assigned when information is absent and a score of 1 is assigned when information is present. SBOSC scores could range from 0-6.

### Data Analyses

Descriptive statistics were calculated for the app features (percentage and number). HONcode scores were derived by summating scores across the 8 criteria, and descriptive statistics were produced (range, mean, SD, median, and IQR). Likewise, SBOSC scores were derived by summating scores across the 6 criteria, and descriptive statistics were produced (range, mean, SD, median, and IQR).

Pearson correlation values between HONcode and SBOSC scores were determined. Variations in HONcode and SBOSC scores with respect to app profile characteristics were determined using the *t* test for independent samples where applicable, using SPSS (version 25.0; IBM Corp).

## Results

The initial search identified 3252 Apps (1074 from the Apple App Store and 2344 from the Google Play Store). Among those identified, approximately one-quarter were duplicates (806/3252, 24.8%), duplicated either within search terms or between platforms. Also excluded were non–English-language (735), non–dental-related (743) and non–oral self-care–related (873) apps. A total of 95 apps were identified as potentially relevant to inform the review and were downloaded (Figure 1). Following the review of the downloaded apps, 54 Apps were identified as relevant to inform the review. Reasons for exclusion included non–age-specific apps (15), inaccessible (after 3 attempts to download) apps (15), non–oral self-care–related apps (6), and apps requiring a product to pair with them (5). A total of 2 independent assessors carried out the search and assessments, and agreement was determined (κ=0.836).

The profile characteristics of the 54 relevant apps are presented in Table 1. Approximately two-thirds of the apps were designed for or targeted children ages 4 years and older (36/54, 67%). Most apps were available on the Apple platform (34/54, 63%) and were free of charge (n=42, 78%). Many of the apps were last updated within the previous 2 years (41/54, 76%) and had a 4-star rating or higher (n=40, 74%). Approximately one-half of the apps were uploaded or developed by a company (26/54, 48%).

HONcode scores ranged from 0-8; approximately one-quarter of apps had a HONcode score of 0 (14/54, 26%) and 4% (n=2) had the maximum score of 8. The mean HONcode score was 1.8 (SD 2.0) and the median score was 1.0 (IQR 0-2.25). SBOSC scores ranged from 0-6; 10 (19%) of the 54 apps had a score of 0 and 2 (4%) had the maximum score of 6. The mean SBOSC score was 1.5 (SD 1.4) and the median score was 1.0 (IQR 1.0-2.0). A summary of HONcode and SBOSC scores is presented in Table 2. There was a significant and positive correlation between HONcode scores and SBOSC scores (*r=*0.37; *P*=.006).

Associations of app profile characteristics with both HONcode and SBOSC scores are presented in Table 3. The target age, platform compatibility, price (payment), and star rating were not significantly associated with HONcode scores (*P*>.05), nor SBOSC scores (*P*>.05). The time since the last update was significantly associated with HONcode scores (*P*=.04), but not SBOSC scores (*P*=.11).

**Table 2:** The number of apps meeting the Health on the Net Foundation Code of Conduct (HONcode) and Scientific Basis of Oral Self-care (SBOSC) criteria (N=54).

Assessment tool and criteria			Apps, n (%)
**HONcode^a^**			
	Authority	4 (7)	
	Complementarity	5 (9)	
	Privacy	11 (20)	
	Attribution	5 (9)	
	Justifiability	13 (24)	
	Transparency	40 (74)	
	Financial disclosure	10 (19)	
	Advertising policy	8 (15)	
**SBOSC^b^**			
	Choice of toothbrush	6 (11)	
	Use of fluoride toothpaste	7 (13)	
	Brushing time	15 (28)	
	Adult supervision	2 (4)	
	Brushing technique	44 (81)	
	Other caries prevention advice	5 (9)	

^a^HONcode: Health on the Net Foundation Code of Conduct.

^b^SBOSC: Scientific Basis of Oral Self-care.

**Table 3:** Variation in the mean Health on the Net Foundation Code of Conduct (HONcode) and Scientific Basis of Oral Self-care (SBOSC) scores with respect to app features (N=54).

Category			HONcode^a^ score, mean (SD)		*P* value^b^		SBOSC^c^ score, mean (SD)		*P* value	
**Age rating in years**										
	3+	1.3 (1.4)		.79		1.4 (0.9)		.26		
	4+	2.0 (2.2)				1.5 (1.6)				
**Compatibility**										
	Android only	1.5 (1.6)		.52		1.3 (0.9)		.44		
	Apple	1.9 (2.2)				1.6 (1.6)				
**Price**										
	Free	1.9 (2.2)		.30		1.4 (1.2)		.39		
	Not free	1.3 (1.3)				1.8 (1.9)				
**Star rating**										
	≥4	1.6 (2.0)		.32		1.6 (1.5)		.44		
	<3	2.1 (2.1)				1.1 (0.9)				
**Last update in years**										
	≤2	2.3 (0.4)		.04		1.5 (1.5)		.11		
	>2	1.1 (0.5)				1.5 (1.1)				
**Producer**										
	Company	1.4 (1.2)		.70		1.8 (1.9)		.92		
	Individual	1.5 (1.6)				1.8 (2.2)				

^a^HONcode: Health on the Net Foundation Code of Conduct.

^b^*P* values are derived from *t* tests for independent samples.

^c^Scientific Basis of Oral Self-care.

## Discussion

### Principal Findings

Over 3000 apps designed for oral self-care among children were identified, and 52 relevant apps informed this review. The quality and scientific basis of these apps were low. More of the new apps were of high quality in terms of scientific basis than older apps.

There has been growing interest in apps promoting general health care and oral health [12-14,17,18]. To follow a systematic approach, the search and identification strategy followed PRISMA (Preferred Reporting Items for Systematic Reviews and Meta-Analyses) guidelines as they are widely used in systematic reviews in the field of dentistry [21]. A broad search strategy was adopted using a wide range of terms for oral hygiene performance. This was also employed in previous systematic reviews, including Cochrane reviews [5-7]. The search was limited to the main mobile app distribution platforms, the Apple App Store and Google Play store (Android), but it is acknowledged that there are other platforms. It was not surprising to have many duplications among search results between platforms as the search strategy had overlapping terms for oral hygiene. Agreement between the 2 independent assessors was high, and where a disagreement occurred, it was resolved through discussion among the supervisors. Thus, there was uniformity in app selection to inform this review.

Most apps were designed for children ages 4 years and older. The current guidelines recommend toothbrushing as soon as the first tooth erupts. There is a need for apps to be developed for a younger age group [20]. It is acknowledged that younger children may not be able to fully comprehend the content of the apps. Nonetheless, the apps can familiarize them and introduce them to the concept of early toothbrushing, as in many other childhood learning apps. Most apps were available on the Apple platform. It was a welcome finding to observe that these apps were mostly free of charge; thus, the potential to use the apps in health promotion and clinical practice is widespread. The majority (approximately three-quarters) of apps were rated 4-stars or above, highlighting the positive feedback from app users.

As previously mentioned, the HONcode is a code of conduct for medical and health websites (including apps). The criteria promote the dissemination of accurate health information through technology and cover 8 principles [19]. In this review, the HONcode scores of the apps varied considerably, with approximately one-quarter having a score of 0 (ie, not following any of the recommendations or guidelines). The overall mean and median HONcode scores were around one-quarter of the total possible score, suggesting that there is room for improvement in enhancing the reliability of app content. The identified apps, in general, had good transparency (40/54, 74%, ie, availability of app developer’s contact information). However, they had low scores for the following criteria: authority (4/54, 7%, ie, having credentialed medical or dental professionals as authors), complementarity (5/54, 9%, ie, stating information to support, not replace, patient–health care professional relationships), and attribution (5/54, 9%, ie, using clear reference sources and indicating when they were last updated). Among the various app profile characteristics, only the time of last update was significantly associated with HONcode scores, in that apps that were uploaded or updated in the past 2 years had higher HONcode scores than those that were uploaded or updated more than 2 years ago. A greater understanding of the need to follow codes of conduct such as the HONcode when publishing health information on websites and social media must be advocated for [19].

For rating the scientific basis of the apps in providing oral health information, the SBOSC, a standardized scale based on 6 criteria for toothbrushing, was used [20]. The 6 criteria were choice of toothbrush, use of fluoride toothpaste, brushing time, adult supervision, brushing technique, and other prevention advice. The SBOSC scores of the apps also varied considerably, with nearly 1 in 5 scoring 0 and few fulfilling all the 6 criteria (less than 1 in 20). The mean and median SBOSC scores were around one-third of the maximum score. This again highlights the need for a massive improvement in the scientific basis of the information provided in apps for oral hygiene.

Interestingly, SBOSC scores and HONcode scores were significantly and positively correlated, although the strength of the correlation could best be interpreted as weak to moderate (*r*<0.5). Thus, apps with high SBOSC and HONcode scores should be promoted. Apps should also be evaluated for their efficacy in enhancing oral hygiene behavior and clinical outcomes related to oral hygiene. In the future, app platforms may consider requesting mHONcode certification (which is HONcode certification specifically for apps) before publishing health care apps to ensure the dissemination of accurate health information. Health care authorities should be encouraged to provide support and funding to professional bodies for developing high-quality oral self-care apps.

### Conclusions

Many apps are available to assist children in adopting oral hygiene practices. The quality and scientific basis of these apps are low. App quality is correlated with its scientific basis, though the strength of the correlation is weak to moderate. Apps updated or developed in the past 2 years are of higher quality than older apps, but there is no evidence that the scientific basis of the apps has improved. There is a need to ensure high-quality and evidence-based apps are available. Their effectiveness in terms of promoting proper oral hygiene behaviors and improving oral health among children should be evaluated.

## Abbreviations

HONcode: Health on the Net Foundation Code of Conduct
PRISMA: Preferred Reporting Items for Systematic Reviews and Meta-Analyses
SBOSC: Scientific Basis of Oral Self-care
